# Gastrectomy reduces pancreatic secretory function via pancreatic atrophy

**DOI:** 10.1007/s00595-023-02685-x

**Published:** 2023-04-21

**Authors:** Shumpei Satoi, Yutaka Kimura, Rie Shimizu, Masataka Matsumoto, Kohei Kawaguchi, Yuta Yoshida, Takaaki Murase, Keiko Kamei, Ippei Matsumoto, Takuya Nakai, Yoshifumi Takeyama

**Affiliations:** Faculty of Medicine, Department of Surgery, Kindai University, 377-2, Ohno-Higashi, Osaka-Sayama, Osaka, 589-8511 Japan

**Keywords:** Gastrectomy, Pancreatic volume, Pancreatic function

## Abstract

**Purpose:**

Although reports suggest that the pancreatic volume decreases after gastrectomy for gastric cancer, the relationship between the pancreatic volume and secretory function after gastrectomy remains unclear. In this study, we examined the relationship between the pancreatic volume and exocrine and endocrine functions after total gastrectomy.

**Methods:**

The pancreatic volumes of 18 distal gastrectomy and 15 total gastrectomy patients were retrospectively measured using computed tomography volumetry up to 5 years postoperatively. Ten low anterior resection patients were selected as controls. In addition, the pancreatic volume and exocrine function evaluated by fecal elastase and the insulin secretory function evaluated by glucagon tolerance testing were prospectively examined before and one year after surgery in nine cases of total gastrectomy.

**Results:**

After low anterior resection, the pancreatic volume did not change, but after distal and total gastrectomy, the pancreatic volume decreased continuously until the fifth year. After total gastrectomy, fecal elastase decreased significantly from 865.8 μg/g to 603.2 μg/g in the first year (p = 0.0316), and the insulin secretion capacity also decreased significantly from 3.83 ng/mL to 2.26 ng/mL (p = 0.0019).

**Conclusions:**

The pancreatic volume decreases continuously after gastrectomy for gastric cancer, and the pancreatic exocrine and endocrine functions decrease along with pancreatic atrophy after total gastrectomy.

## Introduction

Gastric cancer is one of the most common cancers in Japan, and gastrectomy is performed in cases without remote metastatic lesions [1, 2]. After gastrectomy, various postoperative disorders occur, such as meal-related distress of the small stomach, dumping syndrome, diarrhea, and weight loss; therefore, post-gastrectomy syndrome induces a poor quality of life (QOL) [3–5]. Post-gastrectomy syndrome is more severe and occurs more frequently after total gastrectomy (TG) than after distal gastrectomy (DG). In particular, diarrhea and weight loss often occur after TG, partially as a result of pancreatic exocrine insufficiency [6].

The factors affecting the pancreatic exocrine function include pancreatic exocrine stimulation, parenchymal condition, and volume. The pancreatic exocrine function can be evaluated using a secretin load test, a pancreatic function diagnostic (PFD) test, and fecal elastase measurement. After gastrectomy, it has been reported that the pancreatic exocrine function is decreased on loading tests, and fecal elastase levels are decreased as well [6, 7]. However, while it has been reported that the pancreatic volume decreases after gastrectomy [8], there are no reports on the relationship between the pancreatic volume and exocrine or pancreatic endocrine function.

We therefore conducted a retrospective study to clarify the long-term changes in the pancreatic volume after gastrectomy for gastric cancer according to the surgical procedure via a comparison with results obtained following low anterior resection (LAR) for rectal cancer. Furthermore, we prospectively investigated changes in the pancreatic exocrine function by measuring the fecal elastase levels and endocrine function using a glucagon tolerance test in patients who underwent TG.

## Methods

### Retrospective study on changes in PV after surgery

Patients who had undergone DG or TG for gastric cancer between January 2008 and December 2010 were primarily selected in this retrospective study. Patients who had undergone abdominal contrast-enhanced computed tomography (CT) preoperatively and at 1, 3, and 5 years postoperatively and did not meet the following exclusion criteria were selected consecutively for each operation: (i) over 75 years old; (ii) combined resection with the exception of the gallbladder and spleen; (iii) postoperative complications of Clavien–Dindo grade ≥ 2; (iv) postoperative adjuvant chemotherapy; (v) major surgery, relapse, death, or development of other active cancer within 5 years after surgery; (vi) a history of pancreatectomy; and (vii) a history of pancreatic disease, such as chronic pancreatitis, acute pancreatitis, or pancreatic cystic disease, or diabetes. As a result, 18 patients who underwent DG and 15 patients who underwent TG were selected. In addition, 10 patients who underwent LAR for rectal cancer in 2008 were used as a reference group.

Gastrectomy was performed either by open laparotomy or laparoscopically, with D2 or D1 + lymph node dissection. The posterior trunk and hepatic branches of the vagal nerve were dissected in DG and the bilateral vagal nerves were dissected in TG. Reconstruction after DG was performed in a Billroth I or Roux-en-Y fashion, and reconstruction after TG was performed in a Roux-en-Y fashion. LAR for rectal cancer was performed either by open laparotomy or laparoscopically, and reconstruction was performed by rectosigmoid colonic anastomosis using the double-stapling technique.

**Fig. 1:**
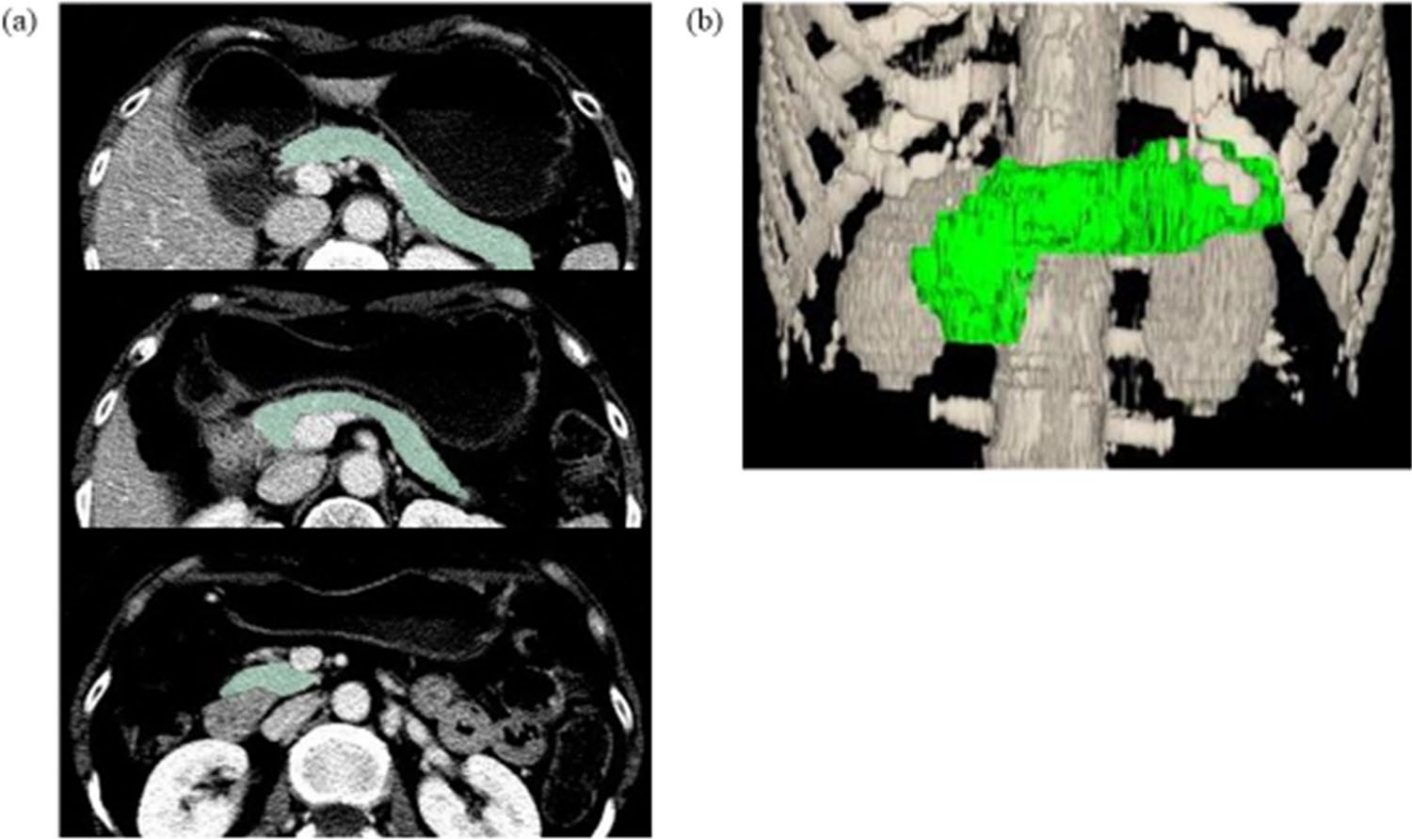
An example of CT volumetry when measuring the pancreas volume. **a** A cross-sectional image of the pancreas, **b** a constructed 3D image of the pancreas

Patients’ data were extracted from medical records, including their age, sex, height, body weight, body mass index (BMI), serum total protein level, serum albumin level, serum total cholesterol level, lymphocyte count, approach of operative procedures, type of operation, reconstruction method, and UICC TNM stage [9]. Pancreatic volume (PV) was measured using an image analysis software program based on CT examinations before and 1, 3, and 5 years after surgery. PV measurements were performed by a radiologist who did not know the patient’s background. The rate of change in the values of clinical data at 1, 3, and 5 years after surgery was calculated by a comparison with the value before surgery. In addition, rectal cancer patients who served as the reference group were measured preoperatively and 5 years postoperatively. PV was measured using the Liver Analysis Application of the SYNAPSE VINCENT® medical imaging system (Fuji Film Medical Corporation, Ltd., Tokyo, Japan) based on 5-mm-slice contrast-enhanced multi detector-row CT data (Fig. 1). Visible pancreatic duct and enhanced vessels were excluded from the PV. Adipose tissue in the pancreas was included in the volumetry.

### Prospective study on the pancreatic function and volume after total gastrectomy

Patients who underwent laparoscopic TG and D1 plus lymph node dissection for early gastric cancer were included in the study. Exclusion criteria were as follows: (i) a performance status of ≥ 2; (ii) likelihood of requiring postoperative adjuvant chemotherapy; (iii) unavailability of contrast-enhanced CT abdominal imaging; (iv) a history of pancreatic disease, such as chronic pancreatitis, acute pancreatitis, or pancreatic cystic disease; and (v) diabetes or endocrine disorders.

PV was measured using an image analysis software program based on CT examinations before and one year after surgery, as previously described. CT attenuation values (Hounsfield Unit: HU) of the pancreatic parenchyma were measured in six different regions of interest (ROIs) by dragging a circular area. The average of four locations was used, excluding the minimum and maximum values. The contour of the pancreas was categorized as either smooth or serrated type. The pancreatic exocrine function was measured using the fecal elastase test. Feces (100 mg) were collected preoperatively and one year postoperatively, and fecal elastase levels were measured using a human elastase-specific antibody enzyme-linked immunosorbent assay (ELISA). An ELISA kit (SK15) from BIOSERV Diagnostics (ScheBo® Biotech AG, Netanyastrasse 3, 35,394 Giessen, Germany) was used. The pancreatic endocrine function was evaluated with a glucagon tolerance test. Glucagon (1 mg) was injected in the fasting state before breakfast. Blood C-peptide levels were measured at the start of the test and 6 min after glucagon injection, and insulin secretion was estimated from the post-loading blood C-peptide level minus the pre-loading blood C-peptide level.

Cross-sectional areas of total skeletal muscle, subcutaneous fat, and visceral fat at the L3 level were measured using the SYNAPSE VINCENT® medical imaging system (Fuji Film Medical Corporation, Ltd.). The BMI, total protein, serum albumin level, total cholesterol level, and total lymphocyte count were measured as nutritional indicators.

Statistical analyses were performed using a statistical software program (JMP, version 16; SAS Institute, Cary, NC, USA). The t-test was used to compare differences between the two groups. The chi-square and Kruskal–Wallis tests were used to compare differences among the three groups. Significance was set at p < 0.05.

This study was approved by the institutional review board of Kindai University Hospital (approval number: 26–245) and was thus performed in accordance with the ethical standards laid down in the 1964 Declaration of Helsinki and its later amendments. Written informed consent was obtained for the prospective study on the pancreatic function, and consent was obtained for the retrospective study via an opt-out method.

## Results

### Retrospective study on changes in PV after surgery

- Patients’ background characteristics

Preoperative patient background characteristics are shown in Table 1. There was no marked difference in the background of patients who underwent the two different surgical procedures, including the age, sex, body weight, BMI, nutritional indicators, surgical approach, and pathological stage of cancer progression.

**Table 1:** Preoperative patient backgrounds for retrospective study

	Total (n = 43)	DG (n = 18)	TG (n = 15)	LAR (n = 10)	p value
Age	63.3 ± 8.1	62.9 ± 8.3	63.1 ± 8.0	64.4 ± 8.5	0.909*
Sex					0.108*
Male	34	17	10	7	
Female	9	1	5	3	
BMI	23.0 ± 3.0	23.9 ± 4.0	22.3 ± 2.2	22.7 ± 1.8	0.524*
pStage					0.805**
pStage I	30	15	12	3	
pStage II/III	13	3	3	7	
Surgical approach					0.458**
Open	16	5	6	5	
Laproscopic	27	13	9	5	
Reconstruction method					
Billroth I	10	10	0	–	
Roux-Y	18	8	10	–	
Nutritional indicator					
Total protein (g/dL)	7.2 ± 0.4	7.1 ± 0.4	7.1 ± 0.4	7.2 ± 0.3	0.765*
Seerum albumin level (g/dL)	4.3 ± 0.3	4.3 ± 0.3	4.3 ± 0.3	4.3 ± 0.2	0.74*
Total lymphocyte count (mm^3^)	2323 ± 862	2330 ± 716	2065 ± 721	2698 ± 1194	0.342*
Pancreas colume (mm^3^)	63.3 ± 20.1	68.3 ± 21.4	54.9 ± 16.6	67.1 ± 20.1	0.203*

* DG vs TG vs LAR

** DG vs TG

**Fig. 2:**
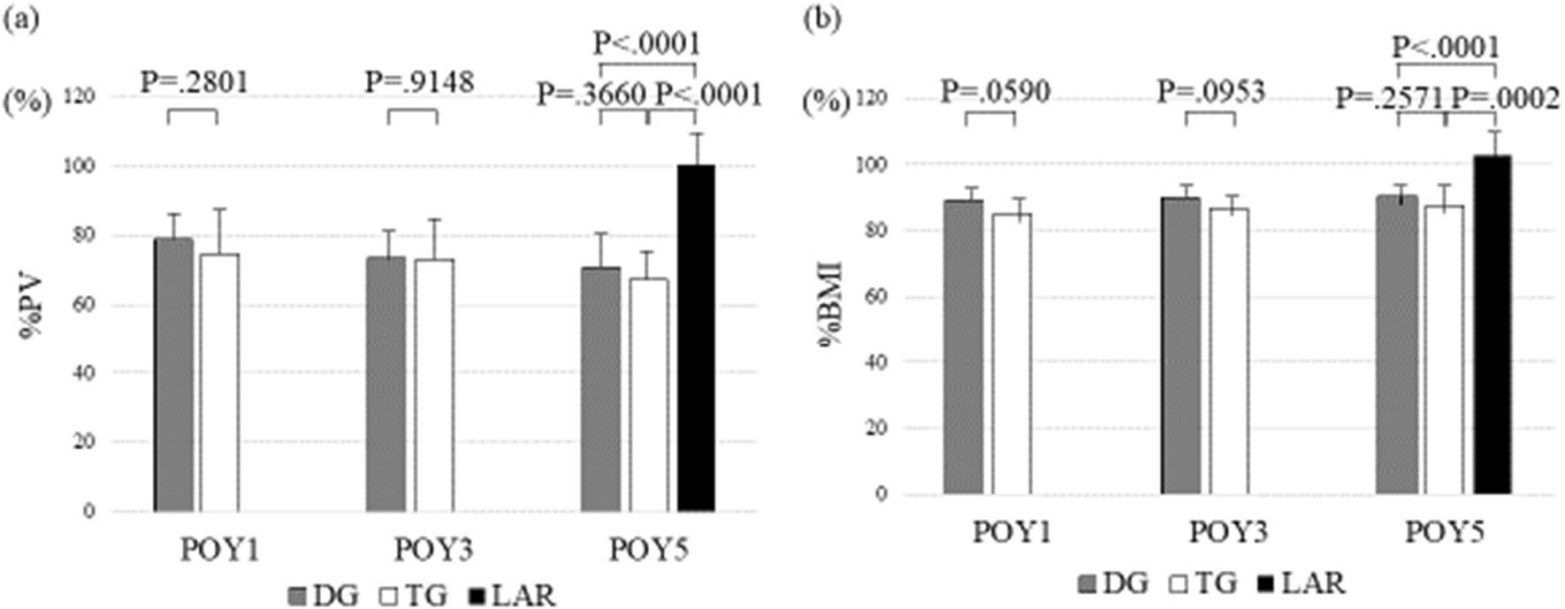
Percentage of the postoperative pancreas volume (PV) relative to the preoperative PV **a** and percentage of the postoperative body mass index (BMI) relative to the preoperative BMI **b** by surgical procedures. %PV, percentage of the postoperative pancreas volume (PV) relative to the preoperative PV; %BMI, percentage of the postoperative body mass index (BMI) relative to the preoperative BMI; *POY* postoperative year, *DG* distal gastrectomy, *TG* total gastrectomy, *LAR* low anterior resection

Figure 1 shows an example of CT volumetry when measuring the PV. Table 1 shows the PV by LAR, DG, and TG. There was no marked difference in the PV between the 3 groups before surgery, but the preoperative PV was positively correlated with the BMI (R^2^ = 0.394, p < 0.0001). The mean pancreatic parenchymal CT values were 43.7 and 44.1 HU pre- and postoperatively, respectively, which was not significantly different (P = 0.283). The contour of the pancreas was the smooth and serrated type in seven and two cases, respectively, with no extreme serrations. Figure 2a shows the percentage of postoperative PV relative to the preoperative PV (%PV) by group. In the LAR group, there was no marked change in the PV between preoperative and 5 years postoperatively, with a %PV of 100.3% at 5 years postoperatively. The PV decreased over time in gastrectomy patients in both the DG and TG groups, and the PV at 5 years postoperatively was significantly lower in both gastrectomy groups than in the LAR group (p < 0.0001). The percentage PV of the DG and TG groups decreased over time: 79.0% and 74.6% (p = 0.2801) at 1 year postoperatively, 73.4% and 73.0% (p = 0.918) at 3 years postoperatively, and 70.6% and 67.4% (p = 0.3660) at 5 years postoperatively, respectively. The PV decreased more evidently in the TG group than in the others, but there was no significant difference between the two gastrectomy groups.

Figure 2b shows the postoperative BMI relative to the preoperative BMI value (%BMI) by group. In the LAR group, there was no marked change in the BMI between preoperatively and 5 years postoperatively, and the %BMI at 5 years postoperatively was 103.0%. In gastrectomy patients, the %BMI at 5 years postoperatively was 90.5% and 87.6% in the DG and TG groups, respectively, which was significantly lower than in the LAR group (p < 0.0002). The %BMI of the DG group remained unchanged from the 1 to 5 years postoperatively, measuring 89.4%, 90.1%, and 90.5% at the first, third, and fifth postoperative years, respectively. Similarly, in the TG group, there was no marked change until the fifth year, with values of 85.2%, 86.9%, and 87.6% at the first, third, and fifth postoperative years, respectively. The %BMI of the TG group tended to be smaller than that of the DG group, but the difference was not significant. Nutritional indicators (serum albumin level and total lymphocyte count) did not differ markedly among groups at 5 years postoperatively (data not shown).

**Fig. 3:**
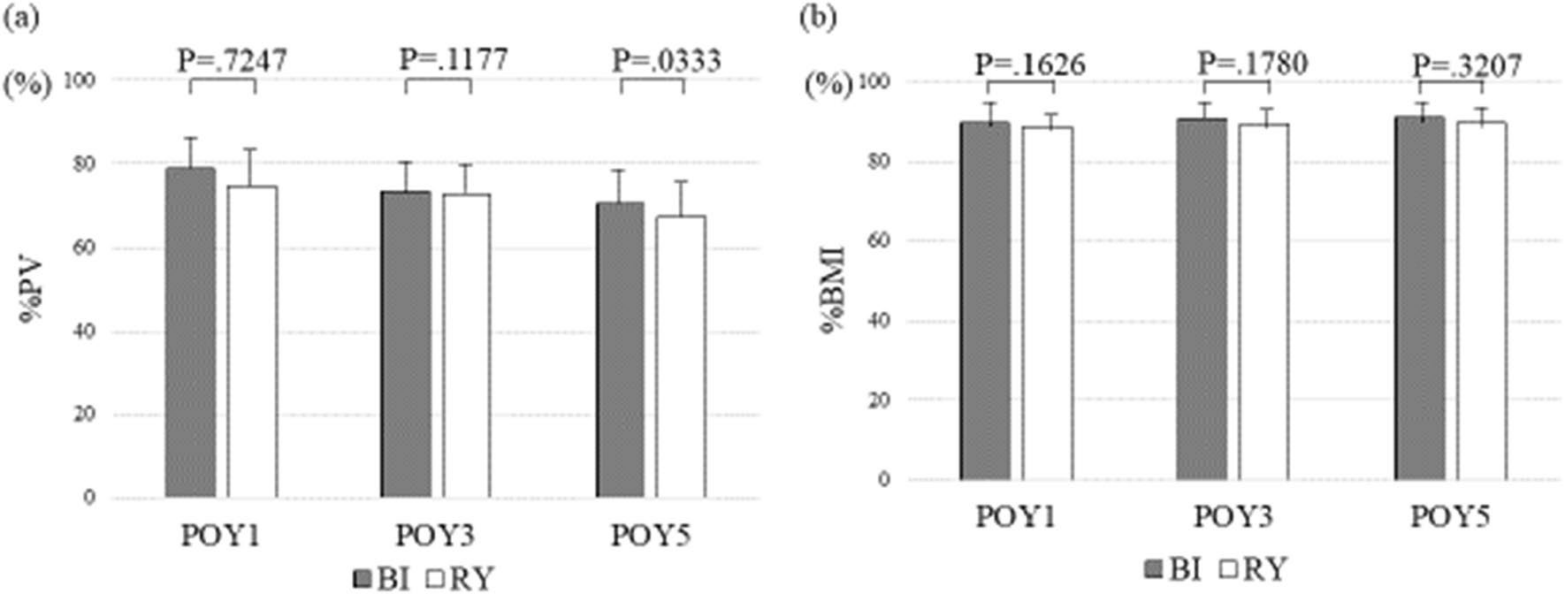
Percentage of the postoperative pancreas volume (PV) relative to the preoperative PV **a** and percentage of the postoperative body mass index (BMI) relative to the preoperative BMI **b** by reconstruction method in cases of distal gastrectomy. %PV, percentage of the postoperative pancreas volume (PV) relative to the preoperative PV; %BMI, percentage of the postoperative body mass index (BMI) relative to the preoperative BMI; *POY* postoperative year, *BI* Billroth I, *RY* Roux-en-Y

The changes in the PV and BMI by reconstruction method in the DG group are shown in Fig. 3. The %BMI did not change in approximately 90% of patients from 1 to 5 years postoperatively for both the Billroth I and Roux-en-Y methods. The %PVs of the Billroth I method were 78.0%, 77.6%, and 74.7% at 1, 3, and 5 years postoperatively, respectively, showing a small change, whereas the Roux-en-Y method tended to show large changes of 80.1%, 68.1%, and 65.5%, respectively.

### Prospective study on the pancreatic function and PV after gastrectomy

Nine patients with early gastric cancer who underwent laparoscopic TG, D1 + lymph node dissection, and Roux-en-Y reconstruction between September 2015 and August 2017 were enrolled in the study. The mean age of the enrolled patients was 72.8 years old, and 6 were male and 3 female. None of the patients had postoperative complications of Clavien–Dindo grade ≥ 2 or had received postoperative adjuvant chemotherapy.

**Fig. 4:**
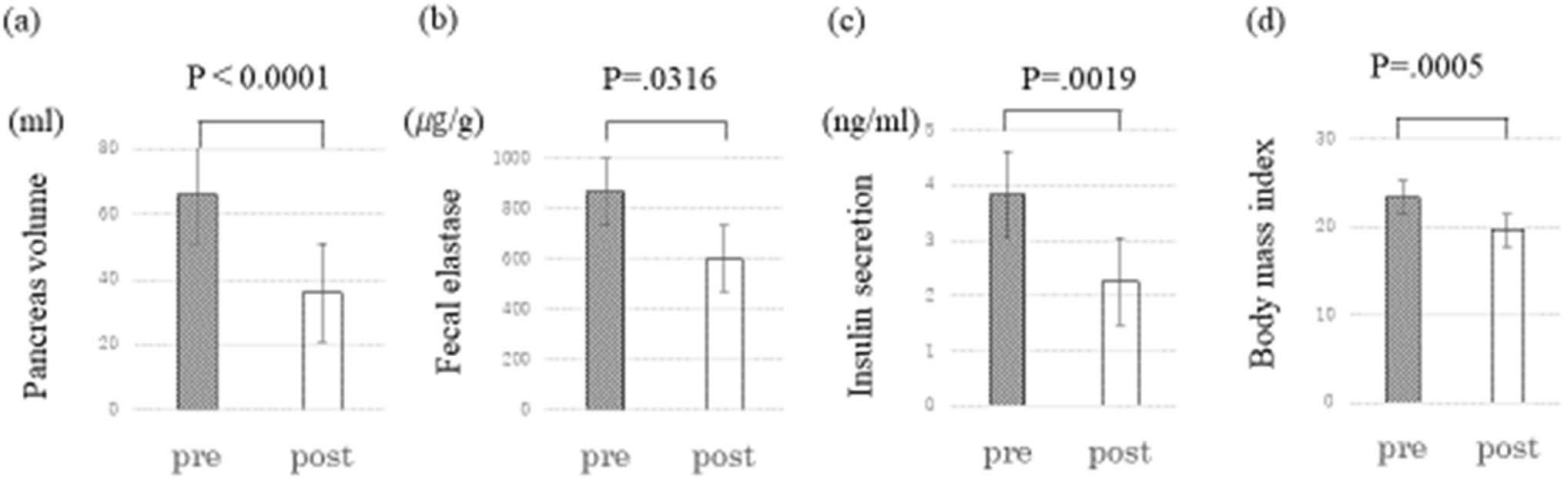
Changes in each parameter before and one year after total gastrectomy. **a** Pancreas volume, **b** fecal elastase, **c** insulin secretion in the glucagon tolerance test, and **d** body mass index. pre, preoperative; post, postoperative

The mean PV was 65.9 mL preoperatively and 36.0 mL in the first postoperative year (p < 0.0001), with a significantly lower %PV of 55.4% in the first postoperative year than preoperatively (Fig. 4a). The mean fecal elastase level was 865.8 μg/g preoperatively and 603.2 μg/g at 1 year postoperatively, indicating a significant decrease (p = 0.0316) (Fig. 4b). The mean insulin secretion in the glucagon tolerance test was significantly lower preoperatively than at the first year postoperatively (3.83 vs. 2.26 ng/mL; p = 0.0019) (Fig. 4c). The mean fasting blood glucose level before the glucagon tolerance test was 104.9 mg/dL preoperatively and 100.1 mg/dL in the first postoperative year (p = 0.1368), and these respective values were 115.1 mg/dL and 115.7 mg/dL after loading (p = 0.44124), showing no significant difference. The mean C-peptide reactivity (CPR) before the glucagon loading test was 1.79 ng/mL preoperatively and 1.46 ng/mL in the first year postoperatively (p = 0.0293), and these respective values were 5.18 ng/mL and 3.47 ng/mL after loading (p = 0.0028). The mean HbA1c was 6.15% preoperatively and 6.00% in the first postoperative year (p = 0.31). We drew a scatter plot of the PV, fecal elastase, and insulin secretion values and found no correlation between the PV and fecal elastase or between the PV and insulin secretion based on preoperative values as well as the rate of decrease.

The mean BMI was 23.4 preoperatively and 19.6 at 1 year postoperatively, with a significant decrease in %BMI of 84.0% at 1 year postoperatively (p = 0.0005) (Fig. 4d). The total skeletal muscle area at the L3 level was 144.6 cm^2^ preoperatively and 128.5 cm^2^ at 1 year postoperatively (p = 0.0095); the subcutaneous fat areas at these points were 105.4 cm^2^ and 53.9 cm^2^, respectively (p = 0.0006); and the visceral fat areas at these points were 107.8 cm^2^ and 25.9 cm^2^, respectively (p = 0.0029), all of which were significantly reduced. The total protein was 7.1 g/dL preoperatively and 6.7 g/dL at 1 year postoperatively (p = 0.0341); the serum albumin levels at these points were 4.3 g/dL and 4.0 g/dL, respectively (p = 0.0171); the total cholesterol levels at these points were 203.0 mg/dL and 173.9 mg/dL, respectively (p = 0.0028); and the total lymphocyte counts at these points were 2,132/m^3^ and 1,700/m^3^, respectively (p = 0.0234), all of which were significantly reduced.

## Discussion

In this study, we demonstrated that PV was reduced after gastrectomy and that not only was the pancreatic exocrine function reduced after TG, but the pancreatic endocrine function was also reduced, along with pancreatic atrophy. The pancreatic secretory function and PV are known to decrease with age [10, 11]. Furthermore, the PV is also decreased in patients with chronic pancreatitis or diabetes in parallel with a decreased pancreatic secretory function [11–13] as well as in patients who undergo laparoscopic sleeve gastrectomy due to severe obesity [14]. Although a decreased pancreatic secretory function and pancreatic atrophy after gastrectomy for gastric cancer have been reported [6–8] separately, the present study clarified for the first time the association between decreased PV and a decreased pancreatic exocrine and endocrine function.

The pancreas is mainly composed of pancreatic parenchyma and fat, and its volume increases up to 20 years old and then remains unchanged until 60 years old, gradually decreasing after that [10]. The higher the BMI, the larger the PV, but also the higher the percentage of fat [10]. The PV is reportedly decreased after gastrectomy, to a greater extent after TG than after DG, and the decrease is greater after Roux-en-Y reconstruction than after Billroth I reconstruction after DG [8]. Although there was no PV change after LAR for rectal cancer, our study showed the same trend after gastrectomy as in previous reports. Since the BMI and PV correlate in healthy adults, we cannot deny the possibility that the PV also decreased as a result of weight loss and nutritional deterioration after gastrectomy. However, the fact that the PV continued to decrease over time until the fifth year after gastrectomy, despite weight loss and nutritional status stabilizing at one year postoperatively, suggests that gastrectomy itself may have had a direct effect on the decrease in PV. Vagotomy and gastrectomy themselves may have influenced the decrease in the PV.

The pancreatic exocrine function was evaluated using the fecal elastase test. Fecal elastase is not easily denatured by refrigeration after stool collection and is used in Western countries as an exocrine pancreatic function test in patients with pancreatic cystic fibrosis and other conditions that cause pancreatic exocrine insufficiency [15]. Secretin loading and C^13^ triglyceride intake studies have previously shown that the pancreatic exocrine function is reduced after TG [6, 16], and in the present study, fecal elastase was approximately 30% lower in the first year postoperatively than preoperatively. Nutritional indicators, such as the BMI, serum albumin level, and total cholesterol level, were significantly decreased but remained within the normal range, and fecal elastase did not decrease to less than 200 μg/g, which indicates pancreatic exocrine insufficiency, and the pancreatic exocrine function in the first year after surgery was not decreased enough to be judged as pancreatic insufficiency.

With the exception of obese patients, the PV and pancreatic function have been reported to correlate in a variety of pathological conditions [10–13], and they were also found to be associated with each other after gastrectomy in this study. Furthermore, the BMI and nutritional indicators as well as skeletal muscle mass, subcutaneous fat, and visceral fat were all shown to be reduced after TG. Weight loss after gastrectomy has been linked to a decreased QOL, decreased compliance with postoperative adjuvant chemotherapy, and worse survival outcomes [17]. If symptoms of suspected pancreatic exocrine hypofunction are present, such as weight loss, diarrhea, or indigestion, using high-titer pancreatic enzyme preparations may be considered in addition to nutritional supplements. [18]. A decreased PV on CT may also be an indirect finding of a decreased pancreatic exocrine function.

The pancreatic endocrine function, or insulin secretion, after gastrectomy varies greatly depending on the timing of insulin measurements. In late dumping syndrome, a typical post-gastrectomy syndrome, excessive insulin secretion is known to occur in response to postprandial hyperglycemia. In contrast, postprandial hyperglycemia in post-gastrectomy patients has also been reported to be due to decreased insulin secretion rather than increased insulin resistance [19]. In the present study, glucagon tolerance test results showed that the insulin secretory ability was decreased after TG, along with the PV and pancreatic exocrine function.

After sleeve gastrectomy for bariatric surgery, PV is thought to decrease due to weight loss and improvement of pancreatic steatosis. The pancreatic exocrine function improves along with the insulin secretory ability [14, 20]. However, after TG for gastric cancer, not only is the body weight and PV reduced, but the insulin secretion and pancreatic exocrine function are also decreased. The difference between the two surgeries is that in sleeve gastrectomy, the vagal nerve is preserved, and food passes through the duodenum, whereas in gastrectomy for gastric cancer, the vagal nerve is resected, and food does not pass through the duodenum after Roux-en-Y reconstruction. It has been reported that Roux-en-Y gastric bypass and biliopancreatic diversion with duodenal switch, which do not pass through the duodenum, are more likely to cause pancreatic exocrine insufficiency than sleeve gastrectomy [21]. Decreased stimulation of the pancreas via the vagal nerve and secretin and cholecystokinin, whose secretion is accelerated when meals pass through the duodenum, may be responsible for the decreased pancreatic secretory function as well as PV after gastrectomy.

Several limitations associated with the present study warrant mention. First, it was a single-center study with a small number of patients, and the pancreatic secretory function was only studied up to the first postoperative year in TG cases. Second, although the quantitative evaluation of PV was performed, there was no qualitative evaluation of the pancreas. Lower CT attenuation values of the pancreatic parenchyma represent fat deposition in the pancreas, but there was no significant difference before and after surgery, and none of the patients in this study had excessive preoperative pancreatic fat deposition. Pancreatic steatosis has been shown to affect the pancreatic secretory function, especially in overweight patients, but no distinction was made between pancreatic fat and parenchyma, and no qualitative evaluation was made. Since the subjects of this study were Japanese gastric cancer patients and not obese patients with a high pancreatic fat content, it is believed that the evaluation was appropriate to some extent. Third, the pancreatic exocrine function was tested by an indirect method using fecal elastase rather than by a loading test. However, the measurement of fecal elastase is a reliable and simple test method for evaluating the pancreatic exocrine function.

In this study, we showed that the PV decreased over time after gastrectomy for gastric cancer, regardless of the surgical type, and that the pancreatic exocrine and endocrine functions decreased along with PV after TG. The mechanism underlying the decrease in the PV after gastrectomy should be studied in the future.

## Data Availability

The data that support the findings of this study are openly available in this article.
